# Knowledge and practice of health extension workers on drug provision for childhood illness in west Gojjam, Amhara, Northwest Ethiopia

**DOI:** 10.1186/s12889-020-08602-y

**Published:** 2020-04-15

**Authors:** Ager Befekadu, Mezgebu Yitayal

**Affiliations:** 1 Felege Hiwot Referral Hospital, Bahir Dar, Amhara National Regional State Ethiopia; 2 Department of Health Systems and Policy, Institute of Public Health, College of Medicine and Health Science, University of Gondar, P. O. Box, 196 Gondar, Ethiopia

**Keywords:** Knowledge, Practice, Health extension workers, Drug provision, Childhood illnesses

## Abstract

**Background:**

The HEP was established decades ago to address preventive, promotive and selective curative services through Health Extension Workers (HEWs). However, knowledge and practice of HEWs on drug provision for childhood illnesses such as diarrhea, fever, and/or acute respiratory infection have not been well studied. This study aimed to assess the knowledge and practice of HEWs on drug provision for childhood illnesses.

**Methods:**

An institutional-based cross-sectional study was conducted among 389 rural HEWs. The districts were selected by using simple random sampling technique, and all the HEWs in the districts were included in the study. Bivariable and multivariable logistic regressions were performed to see the association between knowledge and practice of HEWs on drug provision with the response variables.

**Results:**

The study revealed that 57.5 and 66.8% of HEWs had good knowledge and practice on drug provision for childhood illnesses, respectively. Having college diploma (AOR = 5.59; 95% CI: 1.94, 16.11), 7–9 years (AOR = 2.7; 95% CI: 1.3, 5.5) and 10–12 years (AOR = 2.7; 95% CI: 1.4, 5.4) of experiences, being supervised quarterly (AOR = 0.24; 95% CI: 0.13, 0.47) and biannually (AOR = 0.11; 95% CI: 0.04, 0.30), and having national guideline (AOR = 0.22; 95% CI: 0.06, 0.90) were factors significantly associated with good knowledge. In addition, having college diploma (AOR =3.1; 95% CI: 1.1, 8.8), not receiving refreshment training (AOR = 0.31; 95% CI: 0.11, 0.91), being supervised biannually (AOR = 0.32, 95% CI: 0.13, 0.80), and not having national guideline (AOR = 0.16, 95% CI: 0.04, 0.60) were factors significantly associated with good practice.

**Conclusion:**

The study indicated that a considerable number of HEWs had poor knowledge and practice on drug provision. Socio-demographic factors such as educational status, and work experience; and health systems and support related factors such as training, supervision, and availability of national guidelines, and training had a significant association with HEWs’ knowledge and practice on drug provision. Therefore, designing appropriate strategy and providing refreshment training, and improving supervision and availability of national guidelines for HEWs might improve the knowledge and practice of HEWs on drug provision.

## Background

Evidence shows that 7.2 million children under the age of 5 years globally, and nearly half of it in sub-Saharan Africa lost their lives [1] due to preventable childhood illnesses [2]. Shortage of health workers is more likely to be associated with more childhood deaths, and many countries have implemented task-shifting, a mechanism within which less qualified health workers like community health workers (CHWs) are delegated to provide health services, to deal with the shortage of health workers [3].

The government of Ethiopia launched the Health Extension Program (HEP) in 2003 to extend and expand health service coverage and utilization at the grass-root level, particularly in rural areas. The HEP has four major components (family health services, disease prevention and control, hygiene and environmental sanitation, and education and communication), and 17 HEP packages [4–7]. Simultaneously with the development of the HEP, the government started to train the Health Extension Workers (HEWs) as part of implementation strategies.

The HEWs are mainly females who completed grade 10 and received training on the HEP packages [8]; and they differ from the CHWs since they receive more advanced and comprehensive training and are employed within the government health system and obtain a monthly salary [9]. They spend 75% of their time within the community performing home visits and outreach activities, and 25% of their time at the health posts providing preventive and limited curative services [10]. Currently, there are 42,000 employed HEWs deployed all over the country [11].

One of the four major components of HEP is the family health services. In this component, the HEWs get training on maternal and child health (MCH) care, family planning, immunization, adolescent reproductive health, and nutrition so as to enable them in providing health services to the community within their kebele, the lowest administrative unit within the country [7]. Besides, the national policy change which supports the community-based treatment of childhood pneumonia scaled-up the integrated community case management (iCCM) within the HEP in 2009 [12].

Evidence shows that CHWs are often able to perform health service activities like counseling, health education, behavioral change communication, health promotion, screening, treatment, and referral for diseases [13–15]. In line with this, the implementation of the HEP has demonstrated promising achievements in increasing utilization of family planning, immunization, ANC services, and latrine; and improving knowledge and care-seeking behaviour [10, 16–21]. However, the program did not establish a real success in reducing diarrhea and cough incidences, improving delivery and postnatal care services, and identifying the danger signs and symptoms and complications during pregnancy [10, 22–27]. There is also a high degree of irrational drug prescription such as for antimicrobial drugs [28, 29] that causes significant harm to patients [30], and a claim that CHWs’ lack of knowledge and practice about drugs may increase the inappropriate use of it [31].

The performance and successes of CHWs in prevention, promotion and curative services is influenced by factors associated with gender, marital status, age, and education [32]; and regular and reliable support and supervision [33]. Training, supplies and equipment, supervision, and transport access are the foremost factors that influence the performance of HEWs [34, 35].

A few studies have assessed the HEWs’ knowledge and performance on antenatal care, data management knowledge, and handling of medicine [16, 36, 37]. However, to the best of our knowledge, there is no evidence on the HEWs’ knowledge and practice on drug provision for childhood illness within the study area. This triggered us to see the HEWs’ knowledge and practice on drug provision for childhood illnesses like vaccine given at birth and/or orally, and drugs given for malaria and acute pneumonia. Therefore, this study aimed toward assessing the HEWs’ knowledge and practice on drug provision for childhood illnesses and factors associated with it.

## Methods

### Study design and setting

Institutional based cross-sectional study was conducted in West Gojjam zone to assess the HEWs’ knowledge and practice and associated factors on drug provision for childhood illnesses. The data were collected from February 25–March 30, 2016. The zone is located in the Amhara region, Northwest of Ethiopia. It is divided into 18 districts which are further sub-divided into 393 kebeles (31 urban and 362 rural) kebeles. In this zone, there were 7 Primary Hospitals, 102 Health Centers and 374 Health Posts. According to the 2015 Amhara National Regional Health Bureau report, the total number of HEWs in the Zone was 816 of which 789 HEWs are working in the rural areas.

### Study population and sampling procedures

The study population of this study was all rural HEWs who have been working during the study period. All HEWs in the selected districts were eligible for the study. All voluntary rural HEWs who were working in the selected districts were included in the study whereas HEWs who were on maternity leave and sick leave were excluded from the study.

The sample size was determined using single population proportion formula by taking a proportion of 64% i.e. the proportion of HEWs who provided the correct treatment for children with pneumonia, diarrhea, malaria, and measles [38], 95% CI, 5% margin of error, and 10% non-response rate.

*N* = (Zα/2)^2^*P*(1-p) /w2 = (1.96)2*0.64*(1–0.64)/0.025 = 354.04.

The final sample size was 354.04 + 35.404 (response rate) = 389.44 ≈ 389.

To select the study participants, the investigator purposely selected the West Gojjam zone considering the accessibility of transport and financial feasibility. Among the districts in the zone, seven districts (Yilmana Densa, Mecha, Sekela, Jabi Tehnan, Bahir Dar Zuria, Denbecha, and Debub Achefer) were included in the study. The list HEWs in the selected districts was obtained from the office of West Gojjam Zone. Then, the sample was allocated to the seven districts in which almost all HEWs in the selected districts were included in the study.

### Study variables

#### Response variables

The response variables in this study were knowledge and practice of HEWs on drug provision to childhood illnesses. The overall score was calculated by adding up the scores for each respondent across all questions. Knowledge of HEWs was assessed using 10 questions on basic concepts of drug provision like rational use of drugs, route of administration, and treatment of choice; and HEWs who scored at least 8 points out of 10 were considered as having good knowledge. The practice of HEWs was also assessed using 15 questions like vaccine provision practice, referral practice, and drug control practice; and HEWs who scored at least 12 points out of 15 questions were considered as having good practice.

#### Explanatory variables

The socio-demographic characteristics of the study participants (age, marital status, educational status, religion, and work experience), health systems support (training, supervision, and drug and equipment supply), and organizational characteristics (availability essential drugs, medical equipment, national guideline, drug control systems, and patient registration books) were collected as explanatory variables. Rational drug use was defined as prescribing the right drug, the inadequate dose for the sufficient duration and appropriate to the clinical needs of the patient at the lowest cost. It was measured using a semi-structured questionnaire composed of 8 closed and 2 open-ended questions. The adequacy of training was measured based on length of stay on the job and formal course training, and contents of the childhood treatment training.

### Data collection procedures

Data were collected using a pre-tested and structured questionnaire. The questionnaire was prepared by reviewing HEWs iCCM guideline [13] and reviewing factors related to knowledge and practice in previous studies [12, 16, 36, 37]. The questionnaire addressed questions related to knowledge and practice of HEWs on drug provision for childhood illnesses, and socio-demographic characteristics of the study participants, health system support, and organization characteristics (Supplementary file 1).

For data collection, six supervisors who were BSc nurses and six data collectors who were diploma nurses were trained about the basic techniques of data collection for 2 days. The questionnaire was pre-tested in Semen Achefer and Burie Zuria districts of the West Gojjam zone on 25 HEWs who did not participate in the study, and necessary modification was made. Then, data were collected during HEW’s monthly meeting held at common places near their work area. Such meetings were usually attended by six HEWs from three kebeles. The questionnaire was filled in immediately by HEWs in the presence of data collectors. The collected data were checked by the data collectors’ supervisors as well as the principal investigator.

### Data processing and analysis

The data were entered using Epi info version 7 and exported to SPSS version 20 for analysis. Descriptive statistics were used for organizing, describing and summarizing the data. Both bivariable and multivariable logistic regression was run to determine the association between explanatory variables, and response variables. Initially, bivariable logistic regression was used to identify factors independently associated with the outcome variable at a *p*-value of less than 0.25 based on previous evidence. David W. Hosmer and Stanley Lemeshow in their second edition book entitled “Applied Logistic Regression” recommended using a *P*-value of less than 0.25 as a screening criterion for variable selection for the multivariable analysis [39]. Other published articles used a *p*-value of 0.2 as a cut-off point to select variables for the multivariable analysis [40–43]. Therefore, in this study, variables having *P*-value ≤0.25 in the bivariate analysis were considered for multivariable analysis. Besides, analysis of multicollinearity was performed (Supplementary file 2). Then multivariable logistic regression was used to control the effect of confounding factors. Statistical significance was determined using a 5% level of significance and odds ratio with 95% CI.

## Results

### Socio-demographic characteristics of the respondents

A total of 355 HEWs participated in the study with a response rate of 91.26%. The study revealed that 58% of HEWs aged 18 and 25 years, 56.3% were married, and 51% had a diploma (10 + 3) and 99.7% were Orthodox Christian followers (Table 1).

**Table 1:** Socio-demographic characteristics of HEWs in West Gojjam, Amhara, Ethiopia, 2016

Variables	Category	Frequency	Percent
***Age***	18–25	206	58.0
	26–33	141	39.7
	34–41	6	1.7
	> 42	2	0.6
***Marital status***	Single	137	37.7
	Married	200	56.3
	Windowed	19	5.4
	Divorced	2	0.6
***Educational status***	10 + 1	103	29.0
	10 + 2	23	6.5
	10 + 3	181	51.0
	10 + 4	43	12.1
	Others (12 + 3)	5	1.4
***Religion***	Orthodox	354	99.7
	Muslim	1	0.3
***Work experience in years***	1–3	80	22.5
	4–6	62	17.5
	7–9	87	24.5
	10–12	104	29.3
	Others (less than 1)	22	6.2

### Health system support and organizational characteristics of HEWs

The study revealed that 97.7% of HEWs were ever supervised by supervisors of whom 66.6% were supervised in 3 months interval. More than nine-tenth (93.5%) of HEWs were ever provided additional training on childhood illness of which 39.4% responded that the training provided was not adequate. Among those who responded the training was not adequate, 60.6% of them responded that the training had practical and theoretical problems. The study also revealed that 95.8% of HEWs had reference books or national ICCM guidelines, 93.8% had anti-malaria drugs, and 97.7% had patient registration books in their health posts (Table 2).

**Table 2:** Assessment of health system support and organizational characteristics in West Gojjam, Amhara, Ethiopia, 2016

Variables	Category	Frequency	Percent
***HEWs were ever supervised by supervisors***	Yes	347	97.7
	No	8	2.3
***Frequency of supervision (347)***	1 Month	82	23.6
	3 Months	231	66.6
	6 Months	34	9.8
***Training on childhood treatment***	Yes	332	93.5
	No	23	6.5
***Form of training***^a^	Formal Course	87	24.5
	Workshop/meeting	34	3.4
	On job training	298	83.9
***Training adequacy (332)***	Yes	203	61.1
	No	129	38.9
***Duration of training (332)***	Less than one weak	218	65.7
	One weak	94	28.3
	Two weeks	8	2.
	Other	12	3.6
***Drug supply***	District health office	14	3.9
	Health center	341	96.1
***National guideline***	Yes	340	95.8
	No	15	4.2
***Drugs available in HPs***^a^	ORS	329	92.7
	Anthelmintics	265	74.6
	Anti-malaria	333	93.8
	Amoxicillin	228	64.2
	Cotrimoxazole	214	60.3
	Paracetamol	265	74.6
	Zinc	277	78.0
***Medical equipment***	Yes	339	95.5
	No	16	4.5
***Medical equipment available in HP***^a^	Weigh scale	335	94.4
	Thermometer	318	89.6
	Dressing	179	50.4
	Stethoscope	244	68.7
***Patient register books***	Yes	341	96.1
	No	14	3.9

^a^Multiple responses

### Knowledge and practice of HEWs on drug provision

The study revealed that 57.5% of respondents had good knowledge of drug provision, and 98.9% responded as knowing the vaccine given orally. However, only 39.4% of HEWs responded as knowing the side effects of anti-malaria drugs (Table 3). The study also revealed that 66.8% of HEWs had a good practice on drug provision, and 99.2% responded that they gave OPV immunization. However, only 17.4% of HEWs gave curative services (Table 4).

**Table 3:** Knowledge of HEWs on drug provision in West Gojjam, Amhara, Ethiopia, 2016

Questions		Number	Percent
Child hood illness drug dose prescribe based on	Yes	297	83.7
	No	58	16.3
Vaccine given at birth	Yes	351	98.9
	No	4	1.1
Vaccine given orally for childhood	Yes	350	98.6
	No	5	1.4
First line treatment for uncomplicated Vivax malaria	Yes	340	95.8
	No	15	4.2
Treatment for a child < 5 years with acute pneumonia	Yes	292	82.3
	No	63	17.7
Did you know that anti-malaria drugs have side effect	Yes	220	62.0
	No	135	38.0
Side effect of anti-malaria drugs	Yes	140	39.4
	No	215	60.6
Concepts of rational drug use	Yes	264	74.4
	No	91	25.6
What do you mean by Integrated management of neonatal and child hood illness (ICCM) mean?	Yes	275	77.5
	No	80	22.5
What do you mean by Integrated pharmaceutical Logistics System(IPLS)	Yes	202	56.9
	No	153	43.1
***Overall Score***	***God***	204	57.5
	***Poor***	151	42.5

**Table 4:** Practice of HEWs on drug provision in West Gojjam, Amhara, Ethiopia, 2016

Questions	Responses	Number	Percent
Do you use/refer the national ICCM guideline in your daily activities?	Yes	339	95.5
	No	16	4.5
Do you give health education service about childhood health?	Yes	351	98.9
	No	4	1.1
Do you give BCG immunization service?	Yes	340	95.8
	No	15	4.2
Do you give Measles immunization service?	Yes	345	97.2
	No	10	2.8
Do you give OPV immunization service?	Yes	352	99.2
	No	3	0.8
Do you give Penta immunization service?	Yes	349	98.3
	No	6	1.7
Do you give other Immunization service?	Yes	251	70.7
	No	104	29.3
Do you give curative service for malaria disease?	Yes	339	95.5
	No	6	4.5
Do you give curative service for Pneumonia disease?	Yes	311	87.6
	No	44	12.4
Do you give curative service for diarrhea disease?	Yes	319	89.9
	No	36	10.1
Do you give curative service for parasitic diseases?	Yes	174	49.0
	No	181	51.0
Do you give referral service for serious ill childhood?	Yes	319	89.9
	No	36	10.1
Do you use drug controlling system (Bin card)?	Yes	320	90.1
	No	35	9.9
Do you give other curative services?	Yes	61	17.2
	No	294	82.8
***Overall Score***	***Good***	237	66.8
	***Poor***	118	32.2

### Factors associated with knowledge and practice of HEWs on drug provision

The study revealed that HEWs who had college diplomas (10 + 4) were six times more likely to have good knowledge of drug provision than those who were 10 + 1 level. HEWs who had work experience of 7–9 years and 10–12 years were three times more likely to have good knowledge of drug provision than those who had work experience of 1–3 years. HEWs who were supervised quarterly and biannually were 76 and 89% less likely to have good knowledge compared to HEWs who were supervised monthly, respectively (Table 5).

**Table 5:** Factors associated with knowledge of HEWs on drug provision in West Gojjam, Amhara, Ethiopia, 2016

Variables	Category	Knowledge		COR (95% CI)	AOR (95% CI)
		***Yes***	***No***		
***Education***	10 + 1	56	47	1	1
	10 + 2	12	11	0.92 (0.37, 2.26)	1.2 (0.46, 3.36)
	10 + 3	96	85	0.95 (0.58, 1.54)	1.4 (0.78, 2.44)
	10 + 4	40	8	6.4 (2.3, 17.5)*	5.6 (1., 16.0)*
***Work Experience in yeas***	1–3	32	48	1	1
	4–6	32	30	1.60 (0.82, 3.12)	1.65 (0.8, 3.5)
	7–9	59	28	3.16 (1.68, 5.96)*	2.6 (1.3, 5.4)*
	10–12	73	31	3.53 (1.91, 6.52)*	2.6 (1.3, 5.3)*
	Other	8	14	0.86 (0.32, 2.28)	0.84 (0.24, 2.91)
***Training***	Yes	199	133	1	1
	No	5	18	0.19 (0.07, 0.51)*	0.73 (0.22, 2.34)
***Frequency of supervision***	1 month	67	15	1	1
	3 months	127	106	**0.26 (0.14, 0.49)**	0.24 (0.13, 0.47)*
	6 months	10	24	**0.93 (0.04, 0.24)**	0.11 (0.04, 0.30)*
***National guideline***	Yes	234	106	1	1
	No	3	12	**0.17 (0.05, 0.62)**	0.22 (0.06, 0.90)*

*COR* Crude Odds Ratio; *AOR* Adjusted Odds Ratio; *CI* Confidence Interval, * statistically significant at *P* < 0.05

HEWs who did not receive childhood treatment training were 69% less likely to have a good practice of drug provision compared to HEWs who were trained in childhood treatment. HEWs who were supervised biannually were 68% less likely to have a good practice of drug provision compared to HEWs who were supervised monthly. HEWs who did not have national guidelines were 85% less likely to have a good practice of drug provision compared to HEWs who had national guidelines in their health posts, respectively (Table 6).

**Table 6:** Factors associated with practice of HEWs on drug provision in West Gojjam, Amhara, Ethiopia, 2016

Variables	Category	Practice		COR (95% CI)	AOR (95% CI)
		***Yes***	***No***		
***Age***	18–25	129	77	1	1
	26–33	102	39	1.6 (0.98, 2.48)	1.2 (0.76, 2.05)
***Education***	10 + 1	66	37	1	1
	10 + 2	15	8	1.1 (0.41, 2.71)	1.2 (0.430, 3.46)
	10 + 3	117	64	1.0 (0.61, 1.70)	1.1 (0.65, 1.94)
	10 + 4	38	5	4.3 (1.54, 11.76)*	3.1 (1.08, 8.81)*
***Work Experience in yeas***	1–3	52	28	1	1
	4–6	39	23	0.91 (0.46, 1.82)	0.76 (0.35,1.65)
	7–9	63	24	1.4 (0.73,2.728)	0.97 (0.44, 2.15)
	10–12	76	28	1.5 (0.78, 2.75)	0.73 (0.32, 1.67)
	Other	7	15	0.25 (0.09, 0.69)*	0.28 (0.08, 0.94)*
***Training***	Yes	231	101	1	1
	No	6	17	0.15 (0.006, 0.403)*	0.11 (0.11, 0.90)*
***Supervision***	Yes	236	111		
	No	1	7	0.01 (0.01, 0.55)*	0.41 (0.02, 10.30)
***Frequency of supervision***	1 month	63	19	1	1
	3 months	157	76	0.64 (0.36, 1.2)	0.66 (0.36, 1.21)
	6 months	16	18	0.27 (0.11, 0.62)*	0.32 (0.13, 0.80)*
***National guideline***	Yes	234	106	1	1
	No	3	12	0.11 (0.03, 0.41)*	0.15 (0.04,0.59)*
***Patient registration book***	Yes	231	110		
	No	6	8	0.36 (0.12, 1.05)	0.35 (0.11, 1.10)

*COR* Crude Odds Ratio; *AOR* Adjusted Odds Ratio; *CI* Confidence Interval, * statistically significant at *P* < 0.05

## Discussion

The result of this study showed that 57.5% of HEWs had good knowledge of drug provision for childhood illness. The result is consistent with a study done in the Oromia region among 150 health posts (53%) [38]. Besides, 66.8% of HEWs had a good practice on drug provision for childhood treatments which is in line with the result of a study done in the Oromia region (64%) [44]. However, this study revealed that 42.5% HEWs had poor knowledge and 33.2% of HEWs had poor practice on drug provision for childhood illnesses. This is a considerable magnitude if we take the 42,000 HEWs currently employed in the country into consideration [10] though the study was not assumed to represent the HEWs employed all over the country.

In this study, socio-demographic factors such as educational status, and work experience had a significant association with HEWs’ knowledge and practice on drug provision for childhood illnesses.

The HEWs who had a college diploma (10 + 4) level were more likely to have good knowledge and practice of drug provision than those who had certificate level (10 + 1) education. This result is in line with the study in Kenya and Nigeria that reported that the CHWs’ level of education tend to increase the level of general knowledge and hence positively influence the ability of their performance while a lower level of education is associated with lower delivery of health care service [45, 46]. However, this finding is in contrast with the finding of the study in Western Uganda [47]. This might be due to differences in socio-demographic characteristics of study participants.

The HEWs who had work experience of 7–9 years and 10–12 years were more likely to have good knowledge of drug provision than those who had work experience of 1–3 years. This might be due to HEWs who serve 7–9 and 10–12 years of experience had got better knowledge from their supervisors and the national guidelines than those who serve 1–3 years of experience [48, 49]. In the same token, the current study revealed that 13.5% of HEWs were level-4 and above in educational status, of which 91.7 and 64.9% had work experience of 7 years and above and 64.9% had 10 years and above, respectively.

The study also revealed that health systems and support related factors such as supervision, availability of national guidelines, and training had a significant association with HEWs’ knowledge and practice on drug provision for childhood illnesses.

HEWs who were supervised quarterly and biannually were less likely to have good knowledge of drug provision than those who were supervised in the monthly interval. This result is consistent with a study done in Bangladeshi, and East and Southern Africa in which a high frequency of supervision was associated with a better understanding of working environments of CHWs [14, 33]. HEWs who had no national guidelines in their health posts were less likely to have good knowledge than those who had national guidelines. This is consistent with the findings of the studies done on knowledge and practice of healthcare workers towards infection prevention in healthcare facilities of West Arsi District, and Debre Markos Referral Hospital in Ethiopia in which health facility factors such as availability of guidelines was associated with the knowledge of infection prevention [48, 49],

HEWs who did not receive training were less likely to have a good practice of drug provision than those who were trained in childhood treatment which is in line with a study in Nepal [14]. HEWs who were supervised biannually were less like to have a good practice on drug provision than those who were supervised monthly. This finding is consistent with studies conducted in Uganda and other low-income countries [50, 51]. Besides, HEWs who had no national guidelines were less likely to have a good practice of drug provision than those who had national guidelines. This finding is in line with a study in Addis Ababa [34].

## Limitations

The limitation of this study was that we did not capture data to summarise the reasons for the non-response rate and characterize it by age, sex and kebele and other characteristics such as health system support and organizational characteristics of HEWs; and the use of training to improve HEWs’ knowledge and practice, use of the reference books and ICCM guidelines, and how complete the registration book was. There might also be respondents’ bias as HEWs might tend to give positive responses regarding their knowledge and practice on drug provision for childhood illnesses.

## Conclusion

The study indicated that a considerable number of HEWs had poor knowledge and practice on drug provision. Socio-demographic characteristics of HEWs such as education, and work experience; and health systems and support related factors such as frequency of supervision, training, and availability of national guidelines were factors associated with HEWs’ knowledge and practice on drug provision for childhood illnesses.

Therefore, designing an appropriate strategy to improve education status, improving supervision and availability of national godliness, and providing adequate refreshment training for HEWs focusing on basic concepts and practices of drug provision might improve the knowledge and practice of HEWs on drug provision for childhood illnesses.

## Supplementary information

**Additional file 1.** Questionnaire for assessing the knowledge and practice of HEWs on drug provision for childhood illnessess.

**Additional file 2.** Multicollinearity test of variables for logistic regression.

## Data Availability

The datasets supporting the conclusions of this article are available upon request to the corresponding author. Due to data protection restrictions and participant confidentiality, we do not make participants’ data publicly available.

## Abbreviations

AOR: Adjusted odds ratio
CHWs: Community health workers
HEP: Health extension program
HEWs: Health extension workers
ICCM: Integrated community case management
MCH: Maternal and child health
