# Supplementary material for: Knowledge and practice of health extension workers on drug provision for childhood illness in west Gojjam, Amhara, Northwest Ethiopia
Source: BMC Public Health. 2020 Apr 15;20:496. doi: 10.1186/s12889-020-08602-y (PMC7160904; doi:10.1186/s12889-020-08602-y)
Supplement: Supplementary file 1 — Additional file 1. Questionnaire for assessing the knowledge and practice of HEWs on drug provision for childhood illnessess. [file 12889_2020_8602_MOESM1_ESM.docx]

**Annex 3. Information Sheet and Consent Form**

**Title of the research project:**

Knowledge and Practice of Health Extension Workers on Drug Provision for Childhood Treatment in West Gojjam Zone, Amhara Region

**Information Sheet and Consent Form**

**Name of the Principal Investigators:** Ager Befekadu Sisay

**Name of the Organization:** Institute of Public Health, College of Medicine and Health Sciences, University of Gondar

Information Sheet and Consent Form Prepared for Health extension workers in West Gojjam Zone in seven selected district those are going to participate in this research project.

**Introduction:** This information sheet and consent form is prepared with the aim of assessing the knowledge and practice of health extension workers concerning on drug provision to childhood illness. The research group includes the principal investigator six trained data collectors, six supervisors and one advisor from University of Gondar.

**Purpose:** The aim of this study is to determine knowledge and practice of health service extension program on drug provision and factors associated with drug provision for childhood treatment in West Gojjam Zone in seven selected districts. The selected districts are Yilmana Densa, Mecha, Sekela, Jabi Tehnan, Debub Achefer, Bahir Dar Zuria and Denbecha. This is an interview that targets to assess knowledge and practice of health service extension workers who are working in Yilmana Densa, Mecha, Sekela, Jabi Tehnan, Debub Achefer, Bahir Dar Zuria and Denbecha districts. Your responses are very important, and will be helpful in planning for the necessary support towards improving the rational drug provision of in the health post.

**Procedure:** In order to assess knowledge and practice of health service extension workers on drug provision for childhood health in Yilmana Densa, Mecha, Sekela, Jabi Tehnan, Debub Achefer, Bahir Dar Zuria and Denbecha. We invite you to take part in our project. If you are willing to participate in our project, you need to understand and sign the consent form. Then, you will be asked to give your response by the data collectors. For this questionnaire based study, participants are health service extension workers in Yilmana Densa, Mecha, Sekela, Jabi Tehnan, Debub Achefer, Bahir Dar Zuria and Denbecha selected HEWs. All responses given by the participants, and the results obtained will be kept anonymous and confidential using coding system whereby no one will have access to your responses.

**Risk and/or Discomfort:** By participating in this research project you may feel that it has some discomfort specially on wasting your time but this may not be too much comparing its potential benefits it contributes to the overall understanding the knowledge of health service extension workers on drug provision for treating effectively child health. There is no risk in participating in this research project.

**Benefits:** If you participate in this research project, you may not get direct benefit but your participation is likely to help us in assessing health service extension workers’ knowledge conserving on drug provision for child health in Yilmana Densa, Mecha, Sekela, Jabi Tehnan, Debub Achefer, Bahir Dar Zuria and Denbecha selected HPs, West Gojjam Zone, and Amhara region. It will also give an insight about the health service extension workers drug provision knowledge for child health based on the findings of the study for improving the child health status.

**Incentives:** You will not be provided any incentives to take part in this project.

**Confidentiality and Anonymity:** The information that we will collect from this research project will be kept confidential. Information about you that will be collected from the study will be stored in a file, which will not have your name on it, but a code number assigned to it. Which number belongs to which name will be kept under lock and key, and it will not be revealed to anyone except the principal investigator.

**Right to Refuse or Withdraw**: You have the full right to refuse from participating in this research (you can choose not to respond some or all of the questions) if you do not wish to participate; and this will not affect your health services you get at from any health facilities. You have also the full right to withdraw from this study at any time you wish to, without losing any of your rights as a beneficiary of health services.

**Persons to contact:** If you have any question you can contact any of the following individuals and you may ask at any time you want.

**Name of Principal Investigator:** Ager Befekadu

Tel: **+**251-0945277882**:** E-mail: [agerbefekadu@gmail.com](mailto:agerbefekadu@gmail.com)

**Name of Supervisor:** Dr. Mezgebu Yitayal

Tel: 0947057683

**10. Annex 1. Questionnaires**

**Identification Coding**

| **S.NO** | **Question** |  |
| --- | --- | --- |
| 1 | Date of data collection-------------------------------------------- |  |
| 2 | Questionnaire number--------------------------------------------- |  |
| 3 | Code of data collector----------------------------------------------- |  |
| 4 | Name of Woreda----------------------------------------------------- |  |
| 5 | Identification No of HEWs ----------------------------------- |  |

**Part 1. Questionnaire related to Socio-Demographic and Economic Characteristics**

| **SerNo.** | **Questions** | **Choice of Answer** | **skip** |
| --- | --- | --- | --- |
| 101 | What is your age( in Year) | ______________ Yeas |  |
| 102 | Marital status | 1. Single  2. Married  3. Widowed  4. Divorced |  |
| 103 | What is Your Education Status | 1. 10+1.  2. 10+2  3. 10+3  4.Other(specify)_____ |  |
| 104 | What is your religion | 1. Orthodox  2. Catholic  3. Protestant  4. Muslim  5.Other(specify)____ |  |
| 106 | How long have you been working as  HEW? | 1. 1-3 2. 4-6 3. 7-9 4. 10-12 5. Other(specify) |  |

**Part 2. Question related to Organizational characteristics of health extension workers**

| **Ser.**  **No.** | **Questions** | **Response** | **Skin** |
| --- | --- | --- | --- |
| 201 | Do you have the national guideline for management of neonatal and child hood illness in your HP? | 1. Yes 2. No |  |
| 202 | Are medical equipment; available in your health post | 1. Yes 2. No | If No. 0 skip 204 |
| 203 | What medical equipment’ are available in your health post? (Multiple response is possible) | 1.Weighing scale  2.Thermometer  3.Dressing  4.Stethoscope  5.Other(specify)----------- |  |
| 204 | Do you have register book? | 1. Yes 2. No |  |
| 205 | Do you use drug control system (Bin card)? | 1. Yes  0. No |  |
| 206 | What kinds of drugs available in your health post?  (Multiple response is possible) | 1. ORS 2. Vaccine 3. Anti-hermetic 4. Anti malaria 5. TTC eye ointment 6. Cotrimoxazole Pediatric tab/sy 7. Amoxicillin pediatric tab/sy 8. Paractamole tab/sy 9. Zinc tab 10. Other( specify)- |  |

**Question to assess knowledge of Health Extension workers on drug provision for childhood treatment**

| **Ser.No.** | **Question** | **Response** | |
| --- | --- | --- | --- |
| 301 | How do you decide which drug to prescribe to child hood? | 1. Based on age  2. Based on weight  3. Based on availability of drugs  4. Based on disease type  4.Other (specify)_____________ | |
| 302 | Which vaccine drugs are given at birth? | 1. OPV 2. BCG 3. Measles 4. PCV 5. Penta 6. DPT | |
| 303 | Which vaccine drugs are given orally for childhood? | 1. TT 2. OPV 3. BCG 4. Measles 5. PCV 6. Penta 7. DPT | |
| 304 | What is the first line treatment for uncomplicated vivax malaria for children <5 years? | 1. Chloroquine 2. Coartem | |
| 305 | What would be your recommendation  for a child < 5 years with Acute  Pneumonia? | 1. Amoxicillin  2. Co-trimoxazole  3. Refer the case  4. Advise to take home remedies  5.Other(specify) __________ | |
| 306 | Do you know that anti malaria drugs have side effects? | 1.Yes  0. No | |
| 307 | If your answer is yes, what is the side effect of anti malaria drugs? | ---------------------------------------------------- | |
| 308 | What do you mean rational drug? | | ---------------------------------------------------------------------------------------------------------------------------------------------------------- |
| 309 | Would you explain in your own words what Integrated management of neonatal and child hood illness (ICCM) means? | ---------------------------------------------------------------------------------------------------------------------------------------------------------- | |
| 310 | What do you mean Integrated pharmaceutical Logistics System(IPLS) | --------------------------------------------------------------------------------------------------- | |

**Part 3 Question for assessment of drug provision practice of HEWs**

| ***Ser.***  ***No.*** | ***Questions*** | ***Responses*** | ***Skip*** |
| --- | --- | --- | --- |
|  | Do you use/refer the national guideline in your daily activities | 1. Yes 2. No |  |
| 402 | Do you give health education about child health? | 1. Yes 2. No |  |
| 403 | Do you give curative service for Malaria disease? | 1. Yes 2. No |  |
| 404 | Do you give curative service for Pneumonia disease? | 1. Yes 2. No |  |
| 405 | Do you give curative service for Diarrhea disease? | 1. Yes 2. No |  |
| 406 | Do you give curative service for hookworm disease? | 1. Yes 2. No |  |
| 407 | Do you give curative service for other disease? | 1. Yes 2. No |  |
| 408 | Do you give BCG immunization service? | 1. Yes 2. No |  |
| 409 | Do you give PCV immunization service? | 1. Yes 2. No |  |
| 410 | Do you give Measles immunization service? | 1. Yes 2. No |  |
| 411 | Do you give OPV immunization service? | 1. Yes 2. No |  |
| 412 | Do you give Penta vaccine immunization service? | 1. Yes 2. No |  |
| 413 | Do you give other vaccine service? | 1. Yes 2. No |  |
| 414 | Do you give referral service for sever ill childhood? | 1. Yes 2. No |  |
| 415 | Do you register expired and damaged drugs on bin cared | 1. Yes 2. No |  |

**Part Five. Question related to assessment of health system support**

| ***Ser.***  ***No.*** | ***Questions*** | ***Responses*** | ***Skin*** |
| --- | --- | --- | --- |
| 501 | How you been supervised by supervision? | 1. Yes 2. No |  |
| 502 | If your answer is yes, how often are you supervised? | 1. Every month 2. Every three month 3. Every six month 4. Every year 5. Other (specify)------------- |  |
| 503 | Have you ever been trained in child health treatment? | 1. Yes 2. No |  |
| 504 | If yes, in which form did you received your training? | 1. Formal course 2. Workshop 3. On Job training 4. Other (specify)-------------- |  |
| 505 | How long taken stayed the duration of training? | 1. Less than a week 2. One weak 3. Two weak 4. Other (specify)---------------- |  |
| 506 | Was the training adequate for your work | 1. Yes 2. No |  |
| 507 | If not what do you think is lacking? | 1. Theoretical training 2. Practical training 3. Both 4. Other(specify)---------- |  |
| 508 | Who is providing you frequently drug resupply? | 1. District 2. Health Center 3. NGOs 4. Other (Specify) ------------------ |  |
| 509 | What do you see as a major challenge during provision of drugs for childhood treatment? | ____________________________________________________________________________________ |  |

**Thank you!!!**
