# Supplementary figures and images for: Knowledge and practice of health extension workers on drug provision for childhood illness in west Gojjam, Amhara, Northwest Ethiopia
Source: BMC Public Health. 2020 Apr 15;20:496. doi: 10.1186/s12889-020-08602-y (PMC7160904; doi:10.1186/s12889-020-08602-y)

Multicollinearity Test


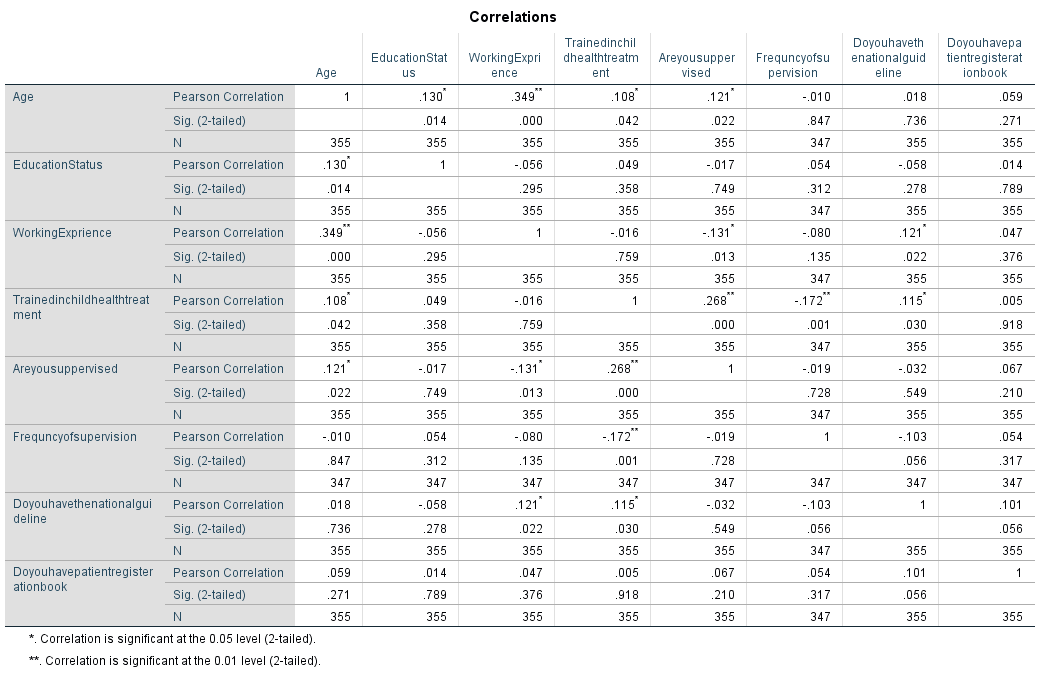

Supplement: Supplementary file 2 — Additional file 2. Multicollinearity test of variables for logistic regression. [file 12889_2020_8602_MOESM2_ESM.docx]
